# Guided Wave and Damage Detection in Composite Laminates Using Different Fiber Optic Sensors

**DOI:** 10.3390/s90504005

**Published:** 2009-05-25

**Authors:** Fucai Li, Hideaki Murayama, Kazuro Kageyama, Takehiro Shirai

**Affiliations:** Department of Systems Innovation, School of Engineering, The University of Tokyo, 7-3-1 Hongo, Bunkyo-ku, Tokyo 113-8656, Japan

**Keywords:** fiber Bragg grating, Doppler effect-based fiber optic sensor, guided wave, composite laminate, damage detection

## Abstract

Guided wave detection using different fiber optic sensors and their applications in damage detection for composite laminates were systematically investigated and compared in this paper. Two types of fiber optic sensors, namely fiber Bragg gratings (FBG) and Doppler effect-based fiber optic (FOD) sensors, were addressed and guided wave detection systems were constructed for both types. Guided waves generated by a piezoelectric transducer were propagated through a quasi-isotropic carbon fiber reinforced plastic (CFRP) laminate and acquired by these fiber optic sensors. Characteristics of these fiber optic sensors in ultrasonic guided wave detection were systematically compared. Results demonstrated that both the FBG and FOD sensors can be applied in guided wave and damage detection for the CFRP laminates. The signal-to-noise ratio (SNR) of guided wave signal captured by an FOD sensor is relatively high in comparison with that of the FBG sensor because of their different physical principles in ultrasonic detection. Further, the FOD sensor is sensitive to the damage-induced fundamental shear horizontal (SH_0_) guided wave that, however, cannot be detected by using the FBG sensor, because the FOD sensor is omnidirectional in ultrasound detection and, in contrast, the FBG sensor is severely direction dependent.

## 1. Introduction

Conventional ultrasonic inspection of large structures is very time-consuming because the transducer needs to be scanned over each point of the structure to be tested. The use of guided waves is potentially a very attractive solution to this problem since they can be excited at one point of the structure and can be propagated over considerable distances [1]. Over the last two decades, ultrasonic guided waves have demonstrated the potential for detecting many defects that occur in tube, pipe or plate structures that are not easily and efficiently detected by other means [2-4]. One major benefit of guided waves is in their rapid global inspection capability. In structural health monitoring (SHM) systems, sensing devices with high sensitivity and accuracy play pivotal roles since damage-contributed ultrasonic guided waves are usually indistinct. So far a number of transducers have been used to capture ultrasonic guided waves in structures. Piezoelectric (PZT) and fiber optic sensors are among the preferred sensors applied in ultrasound detection [5-10], although the electromagnetic interference of the PZT sensor sometimes limits its effectiveness in practical applications [11]. On the other hand, applications of fiber optic sensors are quickly being extended because of their flexibility, high strength, heat resistance, immunity to electromagnetic interference, durability and corrosive resistance [12]. Hence, fiber optic sensors are the most promising among all the currently developed sensors [5] for ultrasound detection purposes.

Although optical interferometric sensors allow sensitive ultrasonic detection, the main drawback of this fiber optic sensor is that a phase control system is required to maintain the optimum sensitivity [13-15]. According to many recent studies, the major focus of interest among the fiber optic sensor community is the fiber Bragg grating (FBG) that has a series of parallel gratings printed onto the core of an optical fiber, and a narrow wavelength range of light is reflected from the sensors when a broadband light is illuminated [16-19]. Since the wavelength at the peak of the reflected signal is proportional to the grating period, the axial strain can be measured through the peak shift [5]. Further, the FBG sensor can be easily multiplexed. Therefore, a number of studies on ultrasonic detection using FBG have been reported in the literature [5,11,15-21]. FBG ultrasonic sensing systems can be classified into two types according to the light source employed. One is a system including a broadband light source and an optical filter [5,17]. An ultrasonic wave can be detected through an optical filter processing of the light reflected from FBG sensor. The other is a system has a tunable laser source in which the intensity of the light reflected from FBG sensor corresponds directly to the ultrasonic response [20,21]. On the other hand, in the authors' previous studies [12,22], a Doppler effect-based fiber optic (FOD) sensor was proposed, which was based on the Doppler effect of light wave transmission in optical fiber and functioned as a vibration/acoustic sensor. Moreover, compared with the FBG sensor, the particular advantages of FOD sensor are: (1) omnidirectional in ultrasonic direction, (2) multiple shapes (such as circular loop, U-shape, and elongated circular loop) that make its use possible in structures with complex geometries, and (3) low cost in manufacturing and constructing an SHM system.

In this paper, two ultrasonic detection systems for the purpose of guided wave and damage detection are presented, which are based on the FBG and FOD sensors, respectively. The FOD sensor is introduced in damage detection for the first time in this study. Section 2 introduces the physical principles of the two types of fiber optic sensors in ultrasonic detection. A piezoelectric transducer was bonded on the surface of a quasi-isotropic carbon fiber reinforced plastic (CFRP) laminate, functioning as actuator to excite Lamb guided waves propagating in the structure. Then in Section 3, by taking advantage of linear-phase finite impulse response (FIR) filter and Hilbert transform, features of guided wave signals are extracted to identify health status of the CFRP laminates by calibrating signal features of an intact CFRP laminate. Further, the extracted signal features were compared to systematically disclose the characteristics of the FBG and FOD sensors in guided wave and damage detection for CFRP laminates. Finally, some conclusions are present in Section 4.

## 2. Guided Wave Detection Using Fiber optic Sensors

### 2.1. Guided Wave Detection Using Fiber Bragg Gratings (FBGs)

An FBG has periodical variation in the refractive index within the core of an optical fiber and acts as a narrowband reflection filter. The central wavelength of light reflected from an FBG is called the Bragg wavelength *λ_B_* and is given by the following equation [11]:

(1) $$\lambda_{B}=2n\Lambda$$

where *n* and *Λ* are the effective refractive index of the fiber core and the grating period, respectively. Under a constant temperature condition, the relative shift in the Bragg wavelength Δ*λ_B_* is in direct proportion to applied strain *ε* along the fiber axis [11]. The shift in the Bragg wavelength is positive when the FBG expands. Conversely, the Bragg wavelength shifts to negative when the FBG shrinks.

**Figure 1.**
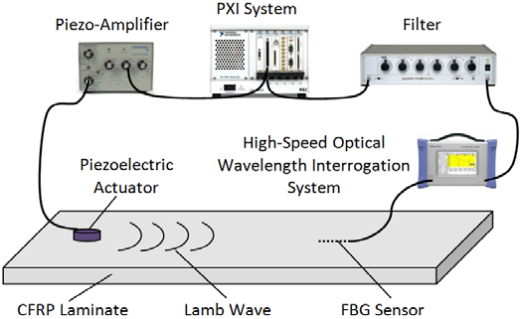
Guided wave and damage detection system using piezoelectric actuator and FBG.

A tunable laser source was used in the present study for guided wave detection. As shown in Figure 1, a PXI-6115 (National Instruments Co., USA) simultaneously functions as an incident wave generator and wave acquisition device. The generated incident wave was amplified by a Piezo-Amplifier (M-2643, MESS-TEK Co., Japan) and was emitted into the specimen by a PRYY-0929 PZT actuator (Physik Instrumente GmbH & Co. KG, Germany) to excite Lamb guided waves. FBG receives guided waves as the strain change of the laminate depending on time and, therefore, center the wavelength of the reflected light from the FBG changes. The wavelength shift is detected using the high speed optical wavelength interrogation system. Finally, the PXI-6115 acquires guided waves filtered by the filter FV-628B (NF Corporation, Japan). FBGs (Fujikura Ltd., Japan, gauge length: 3 mm, wavelength: circa 1,550 nm, full width at half maximum (FWHM): circa 0.5 nm, and reflectivity: > 90%) were used in the present study.

**Figure 2.**
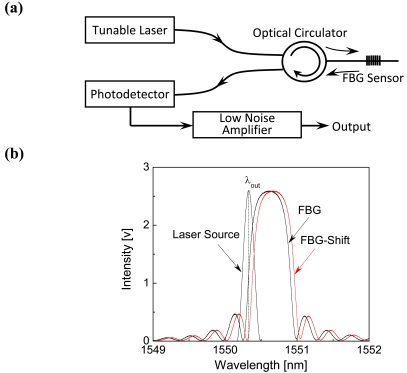
Sketch of the high-speed optical wavelength interrogation system in Figure 1. (a) FBG-based ultrasonic detection system. (b) A schematic illustrating the variation in reflectivity at the lasing wavelength when the FBG sensor expands.

In the ultrasonic detection system with a tunable laser source, the intensity of the light reflected from FBG sensor directly corresponds to ultrasonic response of host structures. A schematic of the high-speed optical wavelength interrogation system, based on tunable laser source, is shown in Figure 2(a). The laser emission wavelength of ‘Tunable Laser’ in Figure 2(a) (Agilent 8164A, Agilent Technologies, USA) is set to *λ_out_* where the reflectivity of the sensor at strain free is reduced by half as shown in Figure 2(b). In this system the optical circulator, photodetector and the low noise amplifier are YC-1100-155 (FDK Corp., Japan), PDA10CS (Thorlabs, USA), and SA-230F5 (NF Corp., Japan), respectively. When FBG expands, the Bragg wavelength shifts to a longer wavelength [‘FBG-Shift’-marked curve in Figure 2(b)] and hence the reflectivity of FBG at the lasing wavelength decreases, and vice versa. In the experiments, gain of the photodetector and optical power of the laser source were 4.75 × 10^3^*V/A* ± 2% (10dB setting) and 2.375 mW, respectively.

### 2.2. Guided Wave Detection Using FOD sensor

The principle of the FOD sensor is based on the Doppler effect of light wave transmission in optical fiber. Consider the light wave, with frequency *f*_0_, transmission in an optical fiber with refractive index *n* and length *L*. When an accident, such as strain rate in host structure of the optical fiber, causes the length of the fiber to change from *L* to *L* + *dL* in an infinitesimal time *dt*, the Doppler frequency shift *f_D_* can be obtained by:

(2) $$f_{D}=-\frac{n}{\lambda_{0}}\cdot \frac{dL}{dt}$$

where *λ*_0_ is the light wavelength in the vacuum, and *λ*_0_/*n* is the light wavelength in the optical fiber.

**Figure 3.**
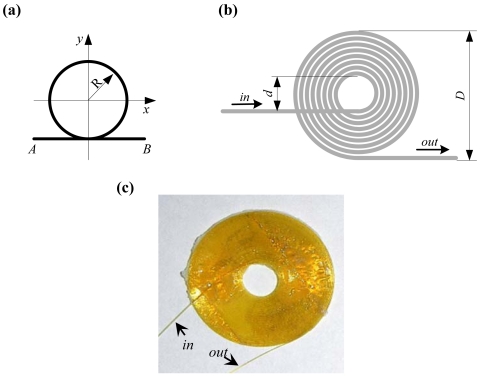
(a) Sketch of circular loop FOD sensor. (b) Sketch of spiral FOD sensor. (c) Picture of the spiral FOD sensor with outer diameter 21.2 mm.

In the previous studies [12,22], three kinds of FOD sensors with different shapes were proposed. The common shape of these FOD sensors is their circular part, as depicted in Figure 3(a), where points *A* and *B* denote the light source and observer, respectively. The theoretical Doppler frequency shift *f_D_* of the circular loop FOD sensor is obtained by [22]:

(3) $$f_{D}=-\frac{\pi Rn_{eq}}{2\lambda_{0}}\left({\dot{\varepsilon }}_{x}+{\dot{\varepsilon }}_{y}\right)$$

where *ε̇_x_* and *ε̇_y_* are the strain rates on *x*- and *y*- directions, respectively, *R* and *D* are the radius and diameter of the circular part of the FOD sensor, respectively, *n_eq_* is the equivalent refractive index of the waveguide and *λ*_0_/*n_eq_* is the equivalent length of light wave in the waveguide. The Doppler frequency shift *f_D_* (or sensitivity of FOD sensor) is directly proportional to the sensing length *L* = *πD* of the FOD sensor. In this study, a spiral shape was selected for the FOD sensor to make it easy to glue it on the surface of CFRP laminate for guided wave detection. A sketch and picture of the spiral FOD sensor are shown in Figures 3(b) and (c), respectively. Heat-resistant optical fiber HEATOP^®^ (Totoku Electric Co., Ltd., Japan) was used to make the spiral FOD sensor in the present study. It is evident the spiral FOD sensor is omnidirectional in ultrasonic detection, which will be discussed in detail in Section 3. Moreover, the FOD sensor can be made manually using only optical fiber and therefore the cost of FOD sensor is considerably smaller than that of FBG.

**Figure 4.**
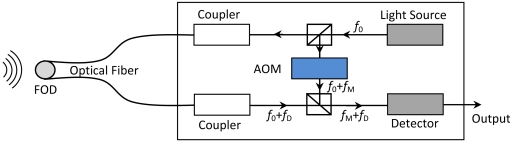
Setup of the laser Doppler velocimeter for the FOD sensor.

A laser Doppler velocimeter (LDV) was used to detect the frequency shift of the FOD sensor, in which extension/compression of the optical fiber was measured. The Doppler frequency shift, *f_D_*, between reflected light from the fiber end and the reference plane, relates to the relative displacement rate (strain rate) *ν* = *dL/dt*. During the detection process, frequency of the reflected laser beam changes from *f*_0_ to *f*_0_ + *f_D_*. The sensitivity depends on the length of the optical fiber bonded on the specimen however the other part of the optical fiber keeps stress free. Setup of the ultrasonic detection system using LDV and the spiral FOD sensor is schematically shown in Figure 4, in which the light source is He-Ne laser (output power: 1 mW, wavelength *λ*_0_: 632.8 nm) and heterodyne interference technique is applied to the measurement. An acoustooptical modulator (AOM) changes the frequency of the reference light source from *f*_0_ to *f*_0_ + *f_M_* (*f_M_* = 80*MHz*) in order to produce beating signals with frequency of *f*_0_ + *f_M_*. A frequency-voltage convertor in the detector is used to offer voltage output for direct ultrasonic acquisition, detailed in Figure 4. Outer diameter, *D*, and inner diameter, *d*, of the spiral FOD sensor used in this study are 21.2 mm and 8.0 mm, respectively. All the FOD sensors used in this study were made by the authors at the University of Tokyo, Japan [Figure 3(c)].

The spiral FOD-based ultrasonic detection system is the same as the FBG-based system except that the high-speed optical wavelength interrogation system in the FBG-based system is substituted by the laser Doppler velocimeter in Figure 4. Moreover, the filter in the FBG-based system (as shown in Figure 1) is necessary because the signal-to-noise ratio (SNR) of acquired guided wave signals using FBG are relatively low [11] and, in contrast, was taken out in the FOD-based ultrasonic detection system [22], which will be discussed in detail in Section 3. Therefore, the cost of the FOD-based system is smaller than that of the FBG-based system without the filter and, moreover, the tunable laser source.

## 3. Guided Wave and Damage Detection in CFRP Laminates

### 3.1. CFRP Laminates

**Figure 5.**
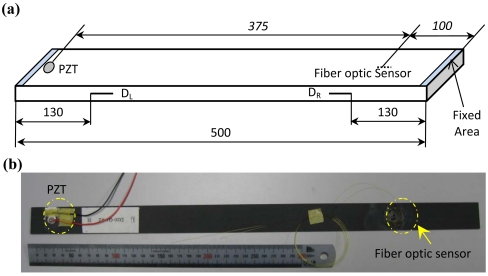
(a) Sketch of the quasi-isotropic CFRP laminate. (b) Picture of a CFRP laminate.

For illustration and without losing generality, quasi-isotropic CFRP laminates are used as host structures for fiber optic sensors-based guided wave and damage detection in the present study. The quasi-isotropic laminates were stacked in accordance with [45/0/-45/90]_2s_ using Pyrofil™ carbon fiber (TR30S, Mitsubishi Rayon Co., Ltd., Japan) with technical properties listed in Table 1. Laminates were fabricated with the dimension of *L* 500 mm × *W* 30 mm × *TH* 1.92 mm. Two types of CFRP laminates were applied in the present study, which are intact and delaminated laminates. Sketch and picture of the specimen are shown in Figures 5(a) and (b), respectively, with the locations of piezoelectric (PZT) actuator, fiber optic sensors and delaminations. In the experiments, the left and the right ends of the specimens were respectively fixed with length of circa 2 mm. For the delaminated CFRP laminates, single- and double-damage scenarios were introduced by inserting one and two thin Teflon^®^ films between the 8^th^ and the 9^th^ laminas of the laminate, respectively. The two delaminations are named D_L_ and D_R_, respectively. For the single-damage laminate, only the D_L_ exists. On the other hand, both the D_L_ and D_R_ are present in the CFRP laminate with two delaminations. To ensure that the guided wave signals are acquired under the same excitation circumstance for the same specimen, experiments for one specimen were done under three procedures: 1) acquiring guided wave signal using FOD sensor; 2) removing the FOD sensor from the specimen and bonding FBG sensor at the same position as the FOD sensor; 3) acquiring guided wave signal using the FBG sensor.

### 3.2. Fiber optic Sensors-Based Damage Detection

In the literature, reflected/transmitted waves were usually used for guided wave-based damage detection [23-25]. In the case of composite structures, delamination could only reflect very faint energy [26] or the reflection could only happen when delamination was introduced in certain plies of the laminates [27]. For example, in the case of semi-infinite delaminated damage, the maximum reflected energy ratio of delamination was less than 0.012, which is relatively small and therefore complicates feature extraction and damage detection using the reflected wave because of noise and the inevitable dispersion. Moreover, when structure is damaged in at least two positions, the problem becomes decidedly more complex. So far relatively few researchers have addressed the multiple damage assessment for structures [28,29]. In the present study, transmitted guided wave is selected for the purpose of fiber optic sensors-based guided wave and damage detection.

#### 3.2.1. Damage Detection Using FBG-Based Guided Signals

**Figure 6.**
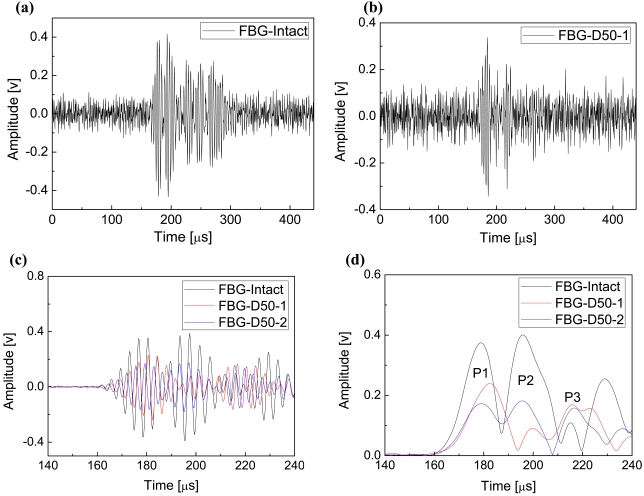
FBG-based results. Guided wave signals of the intact (a) and the single-delaminated (b) CFRP laminates. Waveforms (c) of filtered guided wave signals of the intact and the damaged CFRP laminates and their envelopes (d).

In the present study, a Hanning-windowed 5-cycle sinusoidal toneburst [23-25] at a central frequency of 300 kHz was used as incident signal and guided waves were acquired at sampling rate of 4 MHz. Figures 6(a) and (b) depict the FBG-based guided wave signals captured from the intact CFRP laminate and single-delaminated laminate with the delamination length 50 mm, denoted as ‘FBG-Intact’ and ‘FBG-D50-1’, respectively. Compared with the guided wave signal of intact CFRP laminate, noise dominates the signal of the delaminated laminate, because the delamination depresses the energy of the transmitted guided wave. Moreover, it is evident that it is impossible to identify the locus of each wave package in both Figures 6(a) and (b) because of the strong background noise and superposition of neighboring wave packages. Signal processing techniques are therefore required to offer concise features of the guided wave signals. Since the incident signal was a sinusoidal toneburst at a central frequency of 300 kHz, a bandpass filter was selected to depress the influence of noise and Hilbert transform [30-32] was applied to obtain the envelope of each guided wave signal.

The signal processing algorithm in this study consists of three steps: (1) purifying signals by averaging a number of original guided wave signals; (2) filtering the averaged signal by using bandpass linear-phase finite impulse response (FIR) filter with the passband 100 kHz ∼ 500 kHz; (3) performing Hilbert transform to the filtered signal to obtain its envelope for feature extraction and damage detection. Principles of FIR filter and Hilbert transform are detailed in References [33] and [30-32,34], respectively. Waveforms and envelopes of guided wave signals acquired from the intact and delaminated CFRP laminates are shown in Figures 6(c) and (d), respectively. To reduce the influence of multiple reflection-caused wave packages, only a section of each signal, from 140 μs to 240 μs, is addressed and depicted here.Signal section from 0 μs to 140 μs is noise section, the same as the section from 140 μs to 160 μs, since the transmitted waves did not arrive before around 160 μs; and on the other hand, the signal section after 240 μs has multiple reflection components. Guided wave signals captured from the intact CFRP laminate are used as benchmarks to calibrate the health status of other specimens. In the first step of the above-mentioned signal processing algorithm, the FBG-based guided wave signals were taken average of 60 original signals.

In comparison with the filtered signals in Figure 6(c), it is evident that features of the guided wave signals are more clearly disclosed by taking advantage of Hilbert transform, as shown in Figure 6(d). The legends ‘FBG-Intact’, ‘FBG-D50-1’ and ‘FBG-D50-2’ in Figures 6(c) and (d) denote the FBG-based results of the intact, the single-50 mm-delaminated and the double-50 mm-delaminated CFRP laminates, respectively.

**Figure 7.**
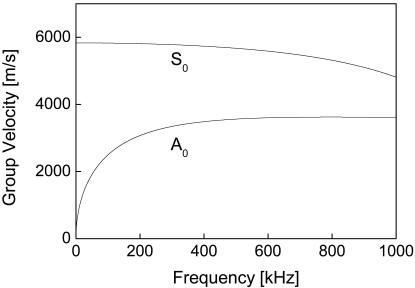
Dispersion curves of the S_0_ and the A_0_ Lamb waves for the quasi-isotropic CFRP laminate.

Envelopes of FBG-based guided wave signals of the intact and the damaged CFRP laminates are conducted, shown as in Figure 6(d), in which each crest denotes one wave package. To ignore the effect of multiple reflection-caused wave packages, only the first three peaks are considered, named P1, P2 and P3, respectively. According to the literature, Lamb waves are a form of elastic perturbation that can propagate in a solid plate. There are two groups of waves, symmetric and asymmetric, that satisfy the wave equation and boundary conditions for this problem and each can propagate independently of the other [24,35]. The fundamental way to describe the propagation of Lamb waves in a particular material is their dispersion curves. The dispersion of these curves begins with the solution to the wave equation for the asymmetric Lamb wave [35]. By using the equivalent mechanics parameters of the quasi-isotropic CFRP laminate [36], the dispersion curves of current CFRP laminates were derived and shown in Figure 7. It is evident that only the fundamental symmetric (S_0_) and the fundamental asymmetric (A_0_) wave modes can be excited under the central incident frequency 300 kHz and theoretical group velocities of the S_0_ and the A_0_ modes are circa 5,880 m·s^-1^ and 3,500 m·s^-1^, respectively.

Considering the distance between the actuator and the fiber optic sensor 375 mm, and the arrival time of the P1 in Figure 6(d), the estimated actual propagating velocity of the wave packages P1 is circa 5,580 m·s^-1^. Therefore, the first wave packages P1 are the transmitted S_0_ Lamb wave. However, it is evident that it is difficult to determine the arrival times of the second wave packages P2 because of the superposition between the wave packages P1 and P2. Therefore, arrival times of peaks of P2 and incident signal were used as substitutes to calculate the actual group velocity of the wave packages P2, which is 3,880 m·s^-1^ as the arrival time of the peak of the incident wave is 99 μs. Hence, the wave packages P2 can be thought the transmitted A_0_ Lamb wave, and the wave packages P3 could be similarly determined, namely the right end-reflected S_0_ Lamb waves.

According to the envelopes in Figure 6(d), all the transmitted S_0_, A_0_ and the right end-reflected S_0_ Lamb waves can be detected using FBG sensor. It is evident that amplitudes of both the transmitted S_0_ and A_0_ Lamb waves (P1 and P2) decrease provided that delamination damages happen in the CFRP laminates. Further, comparing the curves of ‘FBG-D50-1’ and ‘FBG-D50-2’, the second delamination, D_R_ in Figure 5(a), continually decreases the amplitude of the transmitted S_0_. Therefore, those amplitude features of the FBG-based Lamb wave signals can be applied for the purpose of damage detection in the CFRP laminates.

#### 3.2.2. Damage Detection Using FOD-Based Guided Signals

**Figure 8.**
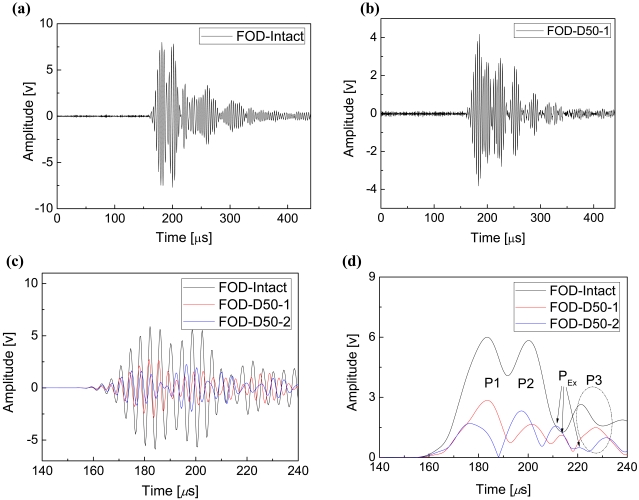
FOD-based results. Guided wave signals of the intact (a) and the single-delaminated (b) CFRP laminates. Waveforms (c) of the filtered guided wave signals of the intact and the damaged CFRP laminates and their Envelopes (d).

FOD-based original guided wave signals of intact and single-delaminated CFRP laminates are shown in Figures 8(a) and (b), respectively. In comparison with the FBG-based signals in Figures 6(a) and (b), it is clear that energy of noise in the FOD-based signals is considerably smaller. Further, delamination-induced amplitude reduction of the signal can be visibly identified from the original signal in Figure 8(b). To offer a better comprehension, the FOD-based guided wave signals were processed using the same signal processing algorithm as the FBG-based signals, in which the FOD-based guided wave signals were taken average of 10 (60 for FBG-based signals) original signals since the signal-to-noise ratios (SNRs) of the FOD-based signals are much larger than that of the FBG-based signals, as shown in Figures 6 and 8. Detail discussion about SNR will be present in Section 3.3. The results are shown in Figures 8(c) and (d). The legends ‘FOD-Intact’, ‘FOD-D50-1’ and ‘FOD-D50-2’ in Figure 8 denote the FOD-based results of the intact CFRP laminate, the laminate with single delamination and double delaminations, respectively.

**Figure 9.**
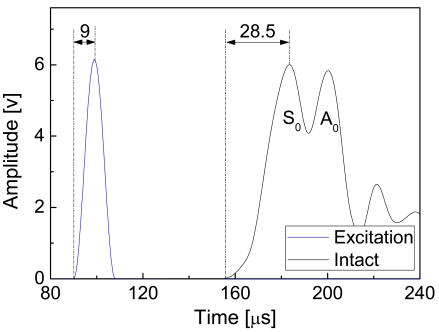
Envelopes of the excitation wave and the guided wave signal captured from the intact CFRP laminate.

Envelopes of the excitation wave and the FOD-based guided wave signal acquired from the intact specimen are shown in Figure 9. Arrival times of the excitation signal and the peak of the excitation signal are 90 μs and 99 μs, respectively. As mentioned before, the group velocity of the wave packages P2 can be estimated using the arrival times of the peaks of the excitation wave and P2 because superposition of wave packages P1 and P2 makes it impossible to determine the exact arrival time of P2. It is noteworthy that precision of localizing a Lamb wave package using its peak is relatively low in comparison with that of using its arrival time because of the intrinsic dispersion of Lamb guided waves. As shown in Figure 9, the interval between the arrival time and the peak of the S_0_ mode becomes 28.5 μs in comparison with 9 μs of the excitation wave.

For the FOD-based signals, same processes as the FBG-based processes were performed, and actual group velocities of the wave packages P1 and P2 are 5,760 m·s^-1^ and 3,700 m·s^-1^, respectively, illustrating that the wave packages P1 and P2 in Figure 8(d) for both the intact and delaminated CFRP laminates are the transmitted S_0_ and A_0_ Lamb waves, respectively. The dash-dot-circled wave packages P3 are the right end-reflected S_0_ Lamb waves. Moreover, similar to the FBG-based results, the delaminations result in reduction in the amplitude of the transmitted S_0_ and A_0_ Lamb waves and the second delamination could further decrease the amplitude of the transmitted S_0_ Lamb waves, as shown in Figure 8(d).

#### 3.2.3. Multiple Damage Detection Using FOD-Based Guided Signals

Moreover, features of the FOD sensor-based results in Figure 8(d) are greatly different from the FBG-based results in Figure 6(d), which is that several extra crests P_Ex_ are present between the transmitted A_0_ Lamb waves P2 and the right end-reflected S_0_ Lamb waves P3 in the envelopes of the FOD-based signals. According to the literature [24,37], when the incident wave propagates in an isotropic beam containing delamination, the shear horizontal (SH) guided wave, traveling in a direction perpendicular to the plane of particle motion, can be converted from the incident Lamb guided wave as a result of the interaction between transmitted Lamb waves and delamination in CFRP laminates [24], so as to stratifying the boundary conditions along the discontinuities. It has also been proved that FBG sensor has directivity in ultrasonic detection [5], which is that FBG is not sensitive to the vibration that is perpendicular to the optical fiber. Since the P_Ex_ waves cannot be detected using the FBG sensors, particle motion should be perpendicular to the direction of wave propagation, which is the characteristic of SH guided wave. Moreover, the CFRP laminates in the present study can be considered quasi-isotropic plates [37]. Therefore, these extra crests P_Ex_ should be the Lamb wave-induced fundamental shear horizontal (SH_0_) guided waves.

**Figure 10.**
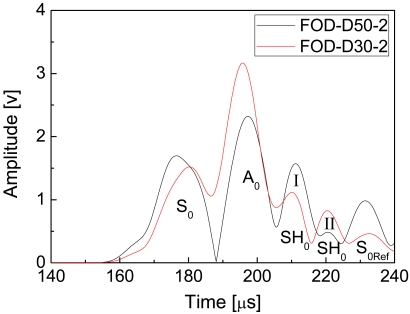
Envelopes of the filtered guided wave signals of CFRP laminates with double-30 mm and double-50 mm delaminations.

Further, there is one extra crest between the P2 and P3 wave packages for the one delamination case in the ‘FOD-D50-1’ curve in Figure 8(d) and, on the other hand, two extra crests are present in the ‘FOD-D50-2’ curve for the two delaminations case. Hence, these extra crests can not only reveal the existence of delamination, but also disclose the number of delamination, which however cannot be offered by using the FBG-based results. Moreover, to verify the proposed results for multiple damage identification, FOD-based guided wave signals were acquired from one double-30 mm-delaminated CFRP laminate. Envelopes of the filtered guided wave signals of CFRP laminates with double-30 mm and double-50 mm delaminations are shown in Figure 10. It is evident that two extra peaks I and II are present in both the envelopes of the double delaminated cases with different damage length.

### 3.3. Discussions

As concluded above, both FBG and FOD sensors can be used for guided wave and damage detection in CFRP laminates. FOD sensor excels FBG sensor in damage detection because it is sensitive to the delamination-induced SH_0_ guided wave that the FBG sensor cannot detect. Based on the principle of ultrasonic detection using FBG in Figure 2(b), it can be concluded that FBG has directivity in ultrasonic detection [5]. In contrast, according to Figure 3, it is evident that FOD sensor is omnidirectional [22] in ultrasonic detection because of its spiral shape and therefore can detect all the propagating guided wave modes in the CFRP laminates.

Moreover, signal-to-noise ratio (SNR) of acquired signal can also indicate performance of a kind of sensor. SNRs of guided wave signals acquired from the intact CFRP laminate using FBG and FOD sensors are listed in Table 2. It is clear that SNR of FBG-based original signal, 26.36, is almost half of that, 52.60, of FOD-based original signal, despite the fact that an extra filter was used in the FBG-based system (as shown in Figure 1). Further, SNR of the average of FBG-based 10 original signals is also much smaller than that of the FOD-based signals, and SNR of the average of FBG-based 60 original signals is even smaller than that of the FOD-based originally acquired signal, viz. 43.68 versus 52.60. Therefore, in comparison with the FBG sensor, the spiral FOD sensor can offer higher SNR in guided wave detection.

**Figure 11.**
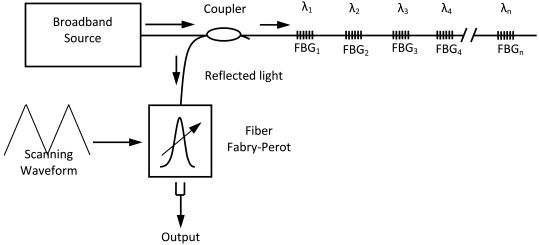
Illustration of scanning filter fiber Bragg grating (FBG) detection technique.

Despite the fact that the FOD sensor exceeds the FBG sensor in omnidirectional properties and ability to offer higher SNR of the acquired guided wave signals, the FBG sensor also has its own particular advantages such as multiplexing. Wavelength division multiplexing (WDM) is one of the major multiplexing arrangements. For illustration, Figure 11 shows a wavelength division multiplexing (WDM) scheme, such as is used with a series of FBGs, each written at a slightly different wavelength, with care being taken to avoid an overlap of the wavelength of one fully perturbed sensor with the spectral envelope of the next. In this way, WDM-based architecture can be the basis of systems where multiple channels may be used to create true multi-sensor systems [38]. In contrast, so far multiplexing technique for FOD sensor has not yet been invented.

## 4. Conclusions

Guided wave and damage detection for carbon fiber reinforced plastic (CFRP) laminate using two types of fiber optic sensors, namely the fiber Bragg grating (FBG) and Doppler effect-based fiber optic (FOD) sensor with spiral shape, were systematically studied and analyzed in this study. Ultrasonic detection systems using the two different fiber optic sensors were proposed. A linear-phase finite impulse response (FIR) filter and Hilbert transform were used to purify the captured guided wave signals and extract signal features for the purpose of damage detection. The results demonstrate that both the FBG and FOD sensors are effective to detect the existence of delamination damages in CFRP laminate by using the amplitude reduction of the processed guided wave signals. Moreover, the FOD sensor could further capture the delamination-induced fundamental shear horizontal (SH_0_) guided waves that the FBG sensor could not, which is because that the spiral FOD sensor is omnidirectional in ultrasonic detection and in contrast the sensitivity of FBG sensor is bonding direction dependent. Further, one of the major advantages of the FBG is that it can be multiplexed, which can help construct an FBG sensor network using a common source and detection system. However, so far a multiplexing technique for the FOD sensor has not yet been invented.

## Figures and Tables

**Table 1.** Technical parameters of the carbon fiber (TR30S).

*(a) Technical parameters of the carbon fiber (TR30S)*									
**Product name**	**Ingredient**		**Modulus [GPa]**		**Possion's ratio [g/m]**			**Density [g/m^3^]**	
TR30S	Carbon fiber		235		0.2			1770	
	Epoxy resin		3.23		0.34			1250	
*(b) Elastic properties for individual lamina*									
***E*_11_ [GPa]**	***E*_22_ [GPa]**	***E*_33_ [GPa]**	***G*_12_ [GPa]**	***G*_13_ [GPa]**	***G*_23_ [GPa]**	***ν*_12_**	***ν*_13_**	***ν*_23_**	**Density [g/m^3^]**
140	9.07	9.07	4.25	4.25	2.94	0.258	0.258	0.39	1560

**Table 2.** Signal-to-noise ratio of original and averaged guided wave signals acquired by different fiber optic sensors from the intact CFRP laminate.

**Fiber optic Sensor**	**Signal-to-Noise Ratio (SNR)**		
	**Original Signal**	**Average of 10 Original Signals**	**Average of 60 Original Signals**
FBG-Sensor	26.36	36.30	43.68
FOD-Sensor	52.60	61.72	--------
